# Maintenance of Hypertensive Hemodynamics Does Not Depend on ROS in Established Experimental Chronic Kidney Disease

**DOI:** 10.1371/journal.pone.0088596

**Published:** 2014-02-12

**Authors:** Diana A. Papazova, Arianne van Koppen, Maarten P. Koeners, Ronald L. Bleys, Marianne C. Verhaar, Jaap A. Joles

**Affiliations:** 1 Department of Nephrology & Hypertension, University Medical Center Utrecht, Utrecht, The Netherlands; 2 Department of Anatomy, University Medical Center Utrecht, Utrecht, The Netherlands; Max-Delbrück Center for Molecular Medicine (MDC), Germany

## Abstract

While the presence of oxidative stress in chronic kidney disease (CKD) is well established, its relation to hypertensive renal hemodynamics remains unclear. We hypothesized that once CKD is established blood pressure and renal vascular resistance (RVR) no longer depend on reactive oxygen species. CKD was induced by bilateral ablation of 2/3 of each kidney. Compared to age-matched, sham-operated controls all ablated rats showed proteinuria, decreased glomerular filtration rate (GFR), more renal damage, higher mean arterial pressure (MAP), RVR and excretion of oxidative stress markers and hydrogen peroxide, while excretion of stable nitric oxide (NO) metabolites tended to decrease. We compared MAP, RVR, GFR and fractional excretion of sodium under baseline and during acute Tempol, PEG-catalase or vehicle infusion in rats with established CKD vs. controls. Tempol caused marked reduction in MAP in controls (96±5 vs.79±4 mmHg, P<0.05) but not in CKD (130±5 vs. 127±6 mmHg). PEG-catalase reduced MAP in both groups (controls: 102±2 vs. 94±4 mmHg, P<0.05; CKD: 118±4 vs. 110±4 mmHg, P<0.05), but did not normalize MAP in CKD rats. Tempol and PEG-catalase slightly decreased RVR in both groups. Fractional excretion of sodium was increased by both Tempol and PEG-catalase in both groups. PEG-catalase decreased TBARS excretion in both groups. In sum, although oxidative stress markers were increased, MAP and RVR did not depend more on oxidative stress in CKD than in controls. Therefore reactive oxygen species appear not to be important direct determinants of hypertensive renal hemodynamics in this model of established CKD.

## Introduction

Chronic kidney disease (CKD) is associated with hypertension. Patients with mild to moderate renal insufficiency have increased levels of oxidative stress [1]–[5] i.e. unfavourable redox balance in which pro-oxidants gain the upper hand over anti-oxidants. This results in a net increase in reactive oxygen species (ROS), leading to cellular and tissue damage. Experimentally increasing ROS (superoxide anion and hydrogen peroxide) in the renal medulla induces hypertension [6], [7].

Several studies support the hypothesis that antioxidants may play an important role in the pathogenesis of chronic renal failure and that antioxidant intervention can slow the progression of renal insufficiency in different experimental models of renal disease [8]. On the other hand, with the notable exception of a single study in hemodialysis patients [9], clinical studies showed no beneficial effects of antioxidants in the CKD population [8], [10], [11].

Tempol (4-hydroxy-2,2,6,6-tetramethyl-piperidine-1-oxyl) is a stable low-molecular-weight (172.25 g/mol) cell-permeable superoxide dismutase (SOD) mimetic that has been used to reduce oxidative injury in cell and animal models. Chronic Tempol administration has been shown to ameliorate oxidative stress and lower arterial pressure in various rat models of hypertension: spontaneously hypertensive rats (SHR) [12], Dahl salt-sensitive rats [13], mineralocorticoid-induced hypertension [14], lead-induced hypertension [15], and erythropoietin-induced hypertension in uremic rats [16]. Acute Tempol administration decreases mean arterial pressure (MAP) and renal vascular resistance (RVR) in SHR [17], [18] and in two-kidney one-clip hypertension [19]. Although in the remnant kidney model, chronic Tempol administration decreases oxidative stress, it has only been shown to prevent or reduce increase of blood pressure for 10–14 days after nephrectomy [20], [21].

Catalase, an H_2_O_2_ detoxifying enzyme, has been shown to prevent hypertension induced by the infusion of H_2_O_2_ in the renal medulla [7]. Polyethylene glycol (PEG)-catalase was preferred to catalase, since the conjugation of catalase with PEG enhances cell association and increases cellular enzyme activity [22]. PEG-catalase prevents the markedly increased vascular and urinary H_2_O_2_ levels and rise in blood pressure in hypertension induced by adenosine receptor blockade [23]. In angiotensin-induced hypertension, although blood pressure was markedly decreased during the first days of PEG-catalase administration, this effect waned after only three days [24].

While the presence of oxidative stress as a feature of CKD is well established, its relation to hypertension and related hemodynamics in CKD has not been systematically addressed. In the current study we hypothesized that ROS are not important determinants of hypertensive renal hemodynamics in long-term, established experimental CKD. To this end we developed a novel bilateral renal ablation model that was staged by the level of proteinuria. In order to differentiate hypertensive effects of superoxide and H_2_O_2_, we studied acute effects of the SOD mimetic Tempol or PEG-catalase on blood pressure (BP) and renal hemodynamics in rats with established CKD and age-matched sham-operated control rats. Furthermore, we investigated the effect of both these interventions on oxidative stress in CKD and control rats.

## Materials and Methods

### Ethics statement

The study protocol was approved by the Utrecht University Committee on Animal Experiments, and conformed to Dutch Law on Laboratory Animal Experiments (DEC number 2010.II.05.097 and DEC number 2012.II.03.053).

### Animals

Male inbred Lewis rats (Lew/CRl), 180–200 g, were purchased from Charles River, Germany and housed in a climate-controlled facility with a 12:12-hour light: dark cycle under standard conditions.

In order to develop established CKD in this strain, the rats were subjected to partial ablation of both kidneys. Via laparotomy under isoflurane anaesthesia (5% induction, 1.5–2% maintenance), branches of both renal arteries were coagulated, resulting in loss of approximately 2/3 of total renal mass in a one-step procedure. Age-matched control rats were sham-operated (CON). All rats received an intramuscular injection of analgesia straight after and one day after surgery (Buprenorphine, 0.05 mg/kg). 24-h urine samples were collected weekly for determination of protein excretion, with the rats in individual metabolic cages while fasting, as described [25]. Blood samples were collected from the tail vein for determination of plasma urea and creatinine. CKD was initially accelerated with N(omega)-nitro-L-arginine (L-NNA), a NO-synthase inhibitor (50 mg/L) in drinking water [11] and the standard powdered chow (CRM-FG; Special Diet Services Ltd., Witham, Essex, UK) was supplemented with 6% NaCl until proteinuria exceeded 200 mg/day after a median of 8 weeks (range: 6–9 weeks). Subsequently L-NNA was withdrawn causing proteinuria to initially fall and subsequently increase slowly as described by Quiroz et al. [26] (data not shown). Terminal experiments were planned within a week when proteinuria exceeded 100 mg/day. This time point was reached after a median of 35 weeks (range: 22–56 weeks). This approach ensured that staging of CKD was similar in all rats. Previously we have shown that proteinuria predicts target organ injury in hypertensive rats [27]. Timing of terminal experiments in sham-operated controls was determined by their age-matched CKD litter mates.

One week prior to termination 24 h urinary excretion of markers of oxidative stress (thiobarbituric acid reactive substances (TBARS), 8-isoprostane (EIA kit, Cayman Chemical, Michigan, USA) and hydrogen peroxide (Amplex Red Hydrogen Peroxide/Peroxidase Assay Kit (Molecular probes, OR, USA)) were measured. Urinary excretion of stable NO metabolites NO_2_ + NO_3_ (NOx) were determined by fluorometric quantification of nitrite content [28]. Rats underwent a terminal measurement under anaesthesia as described. L-NNA, Tempol, PEG-catalase, BSA and Buprenorphine were purchased from Sigma-Aldrich. Isoflurane was purchased from Abbott.

### Terminal experiment protocol

On the day of the experiment the trachea was intubated with a 16-G catheter (Venisystems, Abbocath-T, Abbott, Ireland) under isoflurane anesthesia (5% induction, 1.5–2% maintenance). The femoral artery was cannulated in order to obtain direct measurement of MAP and a Transonic flow probe was placed on the left renal artery to measure renal blood flow (RBF) [17], [29], allowing calculation of renal vascular resistance (RVR: MAP/RBF). Urine was collected allowing measurement of kidney function (glomerular filtration rate, GFR: inulin clearance). During surgery, animals received an intravenous infusion of a 150 mmol/L NaCl solution containing 6% bovine serum albumin (BSA) at a rate of 100 µl/kg/min. Following surgery, the infusion was switched to a 150 mmol/L NaCl solution with 1% BSA, containing inulin for measurement of GFR, which was maintained at the same infusion rate throughout the experiment. Following a 60 min equilibration period, after which both signals were stable, baseline data were collected for 15 min. Thereafter, to investigate renal vascular reactivity we continuously infused the SOD mimetic Tempol (180 µmol/kg/h, CKD n = 6, CON n = 4), PEG-catalase (2000 units/kg/h, CKD n = 8, CON n = 5) or vehicle (NaCl, 0.9% 6 ml/kg/h, CKD n = 4, CON n = 4) after baseline measurements. Following a 45 min equilibration period, after which both signals were stable, intervention data were collected for 15 min. This dose for Tempol was chosen because it has already been shown by others that 72–90 µmol/kg is an effective dose and acute response was very rapid to intravenous Tempol in anaesthetized rat with spontaneous hypertension [30]. A dose of 174 µmol/kg caused a decrease in MAP with more than 30 mmHg and when given in an effective dose (72–90 µmol/kg), Tempol reduced the blood pressure in all hypertensive models with evidence of oxidative stress [31]. Moreover, Tempol administration ameliorated 8-isoprostane excretion in several hypertensive models [32], [33]. Fractional excretions of sodium and potassium (FE Na and FE K) were calculated using standard formulae.

### Oxidative stress protocol

To investigate the effect of antioxidants on oxidative stress in our CKD model, we administered Tempol (180 µmol/kg), PEG-catalase (2000 IU/kg) or vehicle (0.9 % NaCl) iv (tail vein) in a separate cohort of CKD rats. Administration of antioxidant or vehicle was time-matched (between 17:30 and 18:30 h) and followed by collection of urine in metabolic cages overnight. We compared TBARS excretion between age-matched CKD (n = 6) and CON (n = 6) rats, treated in a repeated-design experiment with Tempol, PEG-catalase or vehicle in random sequence.

### Renal morphology

Directly after performing the terminal experiment protocol, rats were sacrificed and tissues were collected and fixed in 4% paraformaldehyde for embedding in paraffin or were snap frozen. Glomerulosclerosis (GS) and tubulo-interstitial injury (TI) were scored on PAS-stained paraffin-embedded slides [34]. Furthermore, endothelial cells in the glomeruli and tubuli were stained with rat endothelial cell antigen (RECA). (RECA)^+^ pixels were counted in glomeruli and tubular fields using ImageJ Software (Rasband, W.S., ImageJ, U.S. National Institutes of Health, Bethesda, MD) [35]. In order to evaluate whether Tempol and PEG-catalase caused changes in thesympathetic nervous system, we performed immunohistochemistry using an antibody against marker for sympathetic nerves: tyrosine hydroxylase (TH) [36]. Snap frozen kidney slices were incubated overnight with anti-TH antibody (P40101-0, Pel-Freez Biologicals, 1∶500).

### Gene expression

To determine whether Tempol and PEG-catalase affected renin-angiotensin system (RAS), gene expression of angiotensin II receptor type 1 (AT1), angiotensin converting enzyme 1 (ACE1) and renin in renal tissue was assessed by qPCR as described [35]. Using the same method we assessed the renal expression of vascular endothelial growth factor (VEGF-A), which is responsible for angiogenesis and endothelial cell proliferation [37]. The following TaqMan Gene Expression Assays (Applied Biosystems) were used : (AT1: Rn01435427_m1), (ACE1:Rn00561094_m1), (renin: Rn00561847_m1), (VEGF-A: Rn00582935_m1), (beta-actin: Rn00667869_m1) and (beta-2-microglobulin: Rn00560865_m1). Cycle time (Ct) values for all genes were normalized for mean Ct-values of beta-actin and beta-2-microglubulin which we previously determined to be the two most stable housekeeping genes for renal tissue for all groups.

### Statistics

Values are expressed as mean ± SEM. Data were compared with unpaired T-test, one way analysis of variance (ANOVA) and two-way ANOVA for repeated measurements when appropriate. Tukey test was used as a post-hoc test (P<0.05).

## Results

### Ablation of 2/3 of each kidney leads to established CKD

**Figure 1:**
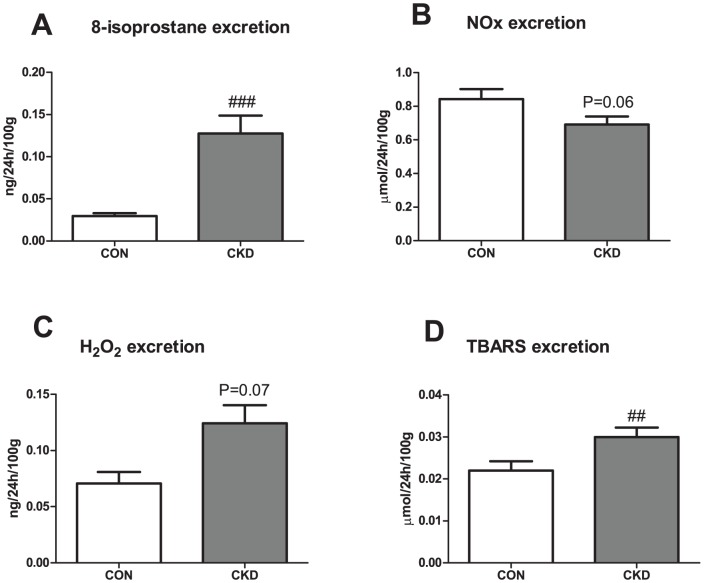
24 h excretions of 8-isoprostanes (panel A), NO metabolites (NOx, panel B), hydrogen peroxide (panel C) and lipid peroxides (TBARS, panel D) in CKD vs. CON rats. Mean ± SEM. ###P<0.001, ##P<0.01 vs. CON.

**Figure 2:**
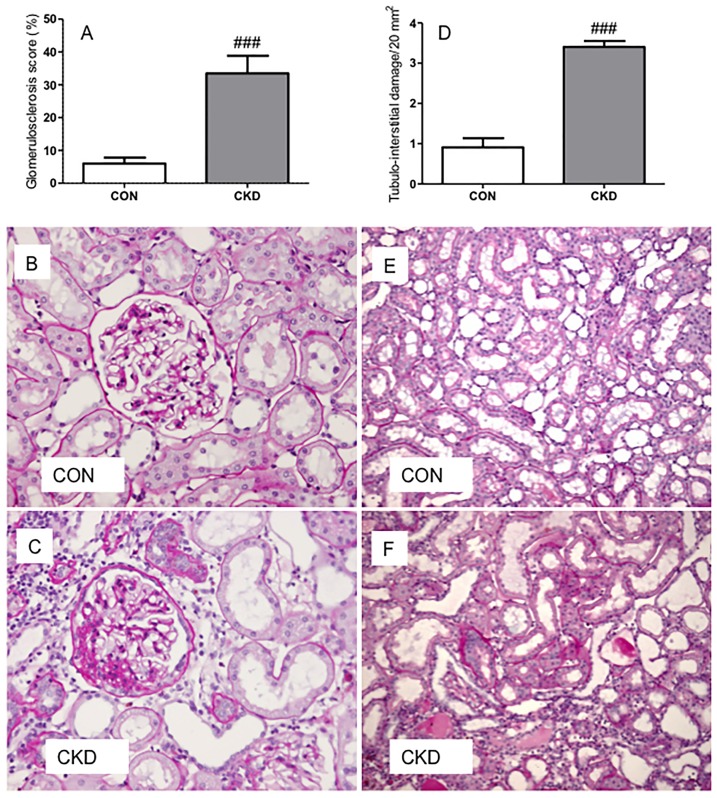
Bilateral ablation (C and F) induced more glomerulosclerosis (panel A) and tubulo-interstitial damage (panel D) in CKD rats compared to controls (B and E) on PAS-stained sections. Means ± SEM. Unpaired t-test: ###P<0.001 vs. CON.

**Figure 3:**
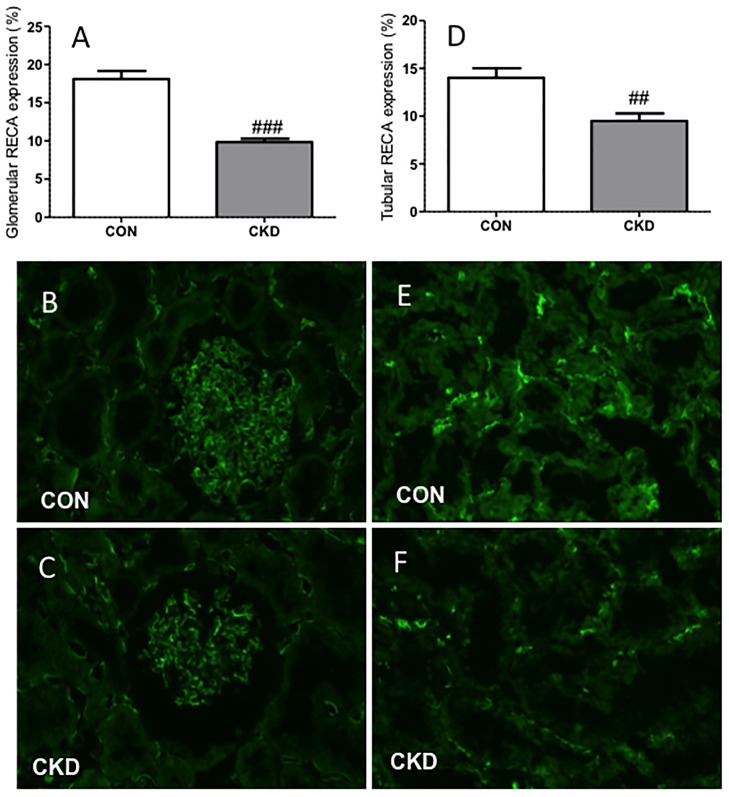
Less (RECA)^+^ pixels (green) were found in CKD rats compared to CON rats in both glomeruli (panel A) and tubular fields (panel D). Immunohistochemical labeling is shown in CON rats (panels B and E) and in CKD rats (panels C and F). Means ± SEM. Unpaired t-test: ###P<0.001; ##P<0.01 vs. CON.

In the CKD group, 3 of the initial 21 animals died during follow-up which resulted in n = 18 of CKD animals, an 85% survival rate. Mortality was either spontaneous (1 rat) or caused by intestinal ischemia in the first week after bilateral ablation possibly due to manipulating the intestines during surgery (2 rats). Survival rate of all sham-operated CON rats was 100%. CKD rats showed slightly lower body weight vs. CON rats (Table 1). All organ weights were corrected for body weight. Renal mass was lower (P<0.01) and heart and wet lungs heavier in CKD rats (P<0.05 and P<0.01 respectively). CKD rats showed increased diuresis (P<0.01) and proteinuria (P<0.001). All CKD rats had mild anemia (P<0.001), higher plasma urea (P<0.001) and creatinine (P<0.05). Markers of oxidative stress were increased in CKD: TBARS and 8-isoprostane excretion were significantly higher (P<0.01 and P<0.001 respectively), whereas H_2_O_2_ excretion tended to increase (P = 0.07) vs. CON rats (Fig. 1). NOx excretion tended to decrease in CKD vs. CON (P = 0.06). In CKD rats, the expression of renin and VEGF-A were lower in comparison to CON rats. No differences in expression of AT1 and ACE1 were found (Table 2). CKD rats showed marked glomerulosclerosis and tubulo-interstitial injury (both P<0.001) (Fig. 2). Counts of RECA-positive pixels indicated lower numbers of endothelial cells in the glomeruli and the tubular fields of CKD rats vs. CON rats (Fig. 3). Visual impression showed no difference in tyrosine hydroxylase expression (Supplemental Figure S1).

**Table 1:** Characterisation of CKD vs. control (CON) rats: organ weights, clinical signs and renal injury.

	CON	CKD
*N*	13	18
Body weight (BW) g	560±14	540±11
Total renal weight (mg/100 g BW)	664±13	591±10 ###
Heart weight (mg/100 g BW)	244±5	280±14 #
Total wet lung weight (mg/100 g BW)	309±6	337±6 ##
		
Diuresis (ml/24 h/100 g)	3.15±0.3	5.45±0.68 #
Proteinuria (mg/24 h)	16±4	152±9###
Hematocrit (%)	45.2±0.3	42.6±0.5 ###
Plasma urea (mmol/L)	6.76±0.18	10.47±0.38 ###
Plasma creatinine (µmol/L)	34±4	50±6 #
Plasma Na (mmol/L)	146.6±1.5	145.7±1.3
Plasma K (mmol/L)	4.25±0.12	4.22±0.07

Mean ± SEM, t-test: # P<0.05, ##P<0.01, ###P<0.001 vs. CON.

**Table 2:** Gene expression of renin, AT1, ACE1 and VEGF-A in CON and CKD rats. Data are presented as log fold change relative to CON.

	CON	CKD
**renin**	0.0±0.37	−1.6±0.35 #
**AT1**	0.0±0.15	−0.7± 0.24
**ACE1**	0.0±0.17	−0.1±0.59
**VEGF-A**	0.0±0.10	−1.4±0.46 #

Means ± SEM. Unpaired T-test. #P<0.05 vs. CON.

### Tempol decreased MAP in CON but not in CKD and did not affect RVR

**Figure 4:**
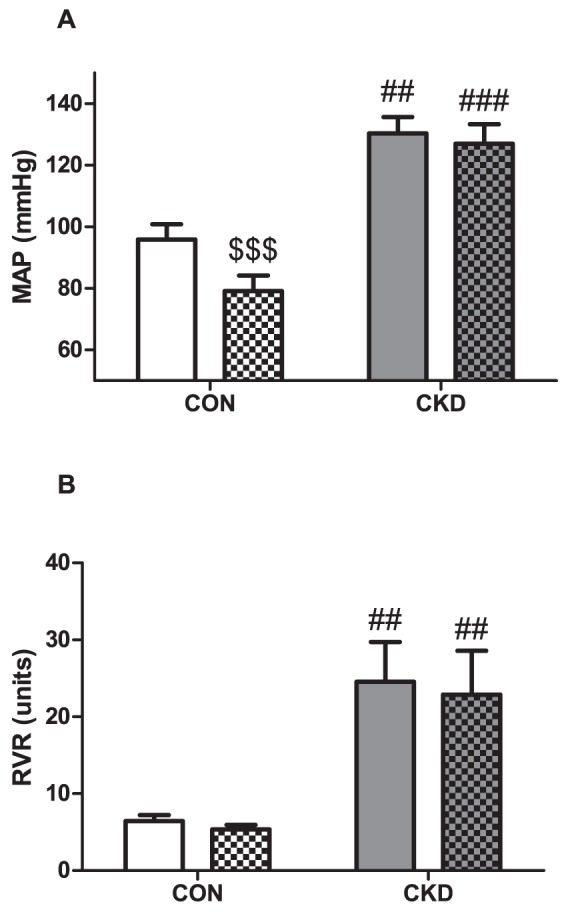
Mean arterial pressure (MAP) (panel A) and renal vascular resistance (RVR) (panel B) prior to baseline (plain bars) and during Tempol (bars with squares) in CON (n = 4, white bars) and CKD (n = 6, grey bars) rats. Mean ± SEM. Two-way RM ANOVA (P CKD vs. CON = 0.001, P Tempol vs. baseline <0.001, P Interaction = 0,002 for panel A; P CKD vs. CON = 0.030, P Tempol vs. baseline = NS, P Interaction = NS for panel B), ## P<0.01, ### P<0.001 vs. CON. $$$ P<0.001 vs. baseline (paired observations).

CKD increased MAP (P = 0.001) and Tempol decreased MAP (P<0.001, Fig. 4A). However, the effect of Tempol was different in CKD than in CON, resulting in strong interaction (P<0.01), and when individual groups were compared with the post-hoc test, we found that infusion of Tempol significantly decreased MAP in CON (P<0.001) but had no effect on MAP in CKD. All CKD rats had higher RVR vs. CON rats (P<0.01) and Tempol had no significant effect on RVR (Fig. 4B).

### PEG-catalase decreased MAP and RVR in CON and CKD

**Figure 5:**
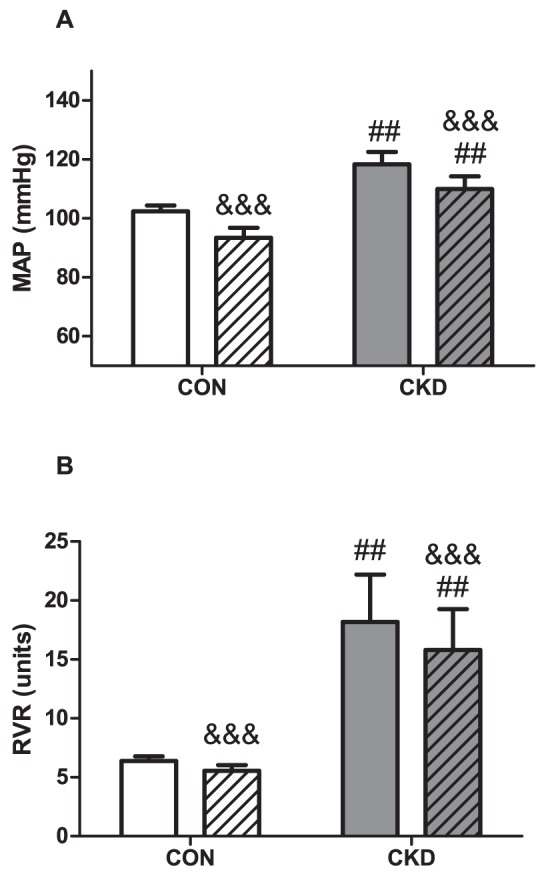
Mean arterial pressure (MAP) (panel A) and renal vascular resistance (RVR) (panel B) prior to baseline (plain bars) and during PEG-catalase (striped bars) in CON (n = 5, white bars) and CKD (n = 8, grey bars) rats. Mean ± SEM. Two-way RM ANOVA (P CKD vs. CON = 0.017, P PEG-catalase vs. baseline <0.001, P Interaction  =  NS for panel A; P CKD vs. CON = 0.001, P PEG-catalase vs. baseline <0.001, P Interaction = NS for panel B). ## P<0.01, ### P<0.001 vs. CON. && P<0.01, &&& P<0.001 vs. baseline (paired observations).

PEG-catalase significantly reduced MAP (P<0.001) in both CON and CKD, and in the post-hoc analysis, PEG-catalase-induced reductions in MAP were all significant (Fig. 5A). For RVR, the same pattern was observed: PEG-catalase decreased RVR in both CON and CKD (P<0.001), and in the post-hoc analysis all PEG-catalase-induced reductions in RVR were significant (Fig. 5B).

### Vehicle

**Figure 6:**
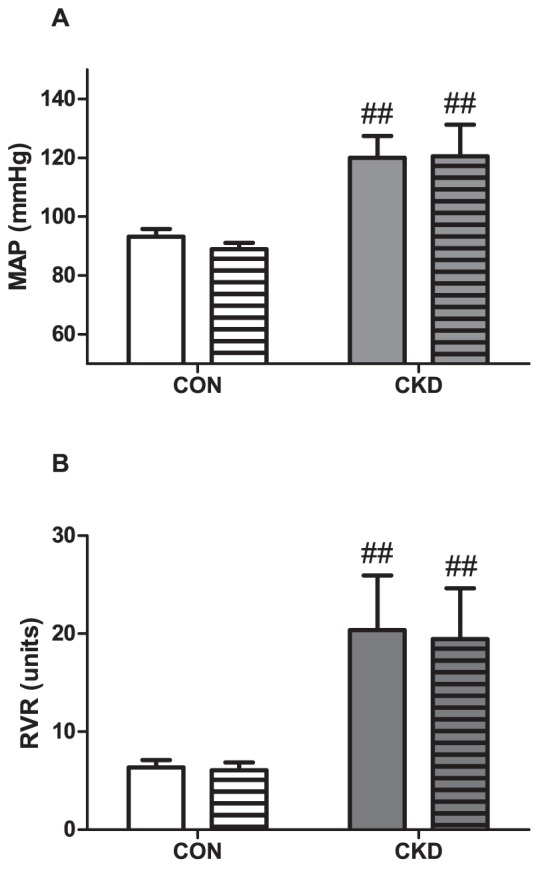
Mean arterial pressure (MAP) (panel A) and renal vascular resistance (RVR) (panel B) prior to baseline (plain bars) and during infusion of vehicle (bars with horizontal lines) in CON (n = 4, white bars) and CKD (n = 4, grey bars) rats. Mean ± SEM. Two-way RM ANOVA (P CKD vs. CON = 0.020, P vehicle vs. baseline  =  NS, P Interaction  =  NS for panel A; P CKD vs. CON = 0.040, P vehicle vs. baseline  =  NS, P Interaction  = NS for panel B). ## P<0.01 vs. CON.

Infusion of vehicle (0.9 % NaCl) did not affect either MAP (Fig. 6A) or RVR (Fig 6B).

### Tempol and PEG-catalase reduced GFR in CON and increased FE Na in CKD

CKD rats had lower GFR vs. CON rats (P<0.001, Table 3). Tempol had different effects on GFR in CKD and CON, resulting in interaction (P<0.05), and when groups were compared with the post-hoc test, Tempol markedly reduced GFR in CON (P<0.001), but not in CKD. Similarly, PEG-catalase had different effects on GFR in CKD and CON (P<0.05), and reduced GFR in CON (P<0.001), but not in CKD. This pattern for GFR was also observed during vehicle infusion.

**Table 3:** Glomerular filtration rate (GFR) and fractional electrolyte excretion prior to (baseline) and after intervention (Tempol, PEG-catalase, vehicle).

	CON		CKD		P-value		
	**baseline**	**Tempol**	**baseline**	**Tempol**	**CKD-cat**	**Tempol**	**Interaction**
*N*	4		6				
GFR (µl/min/100g)	672±65	485±60 $$	302±18 ###	262±21 ##	<0.001	= 0.003	= 0.029
FE Na (%)	0.28±0.12	0.23±0.13	1.02±0.26	1.91±0.54 # $$	= 0.042	= 0.066	= 0.043
FE K (%)	26.09±1.94	25.11±0.73	64.22±4.61 ###	62.43±3.90 ###	<0.001	= 0.665	= 0.898
	**baseline**	**PEG-catalase**	**baseline**	**PEG-catalase**	**CKD**	**PEG-catalase**	**Interaction**
*N*	5		8				
GFR (µl/min/100g)	784±105	510±112 &&&	354±40 ###	273±24 ##	<0.001	<0.001	= 0.021
FE Na (%)	0.15±0.04	0.12±0.04	0.47±0.08	0.79±0.14 ### &&&	= 0.005	= 0.026	= 0.009
FE K (%)	30.47±3.53	28.67±3.56	63.42±5.41 ###	60.96±4.98 ###	<0.001	= 0.501	= 0.915
	**baseline**	**vehicle**	**baseline**	**vehicle**	**CKD**	**vehicle**	**Interaction**
*N*	4		4				
GFR (µl/min/100g)	741±102.13	523±107 §	303±32 ##	240±28 #	= 0.013	= 0.007	= 0.066
FE Na (%)	0.17±0.04	0.16±0.05	1.27±0.60	1.60±0.92	= 0.145	= 0.364	= 0.342
FE K (%)	30.67±0.79	26.77±1.94	73.50 ±8.58 ##	73.45±7.64 ##	= 0.001	= 0.273	= 0.284

Mean ± SEM, ANOVA RM, Tukey post-hoc test for comparison between groups ### P<0.001, ## P<0.01, #P<0.05 vs. CON; $$P<0.01 Tempol vs. baseline; &&& P<0.001 PEG-catalase vs. baseline; § P<0.05 vehicle vs. baseline.

CKD rats had higher FE Na (P<0.01) and FE K (P<0.001) than CON rats (Table 3). During Tempol infusion FE Na tended to decrease in CON (NS), but was markedly increased in CKD (P<0.01), both compared to their own baseline. This pattern resulted in a significant interaction for FE Na (P<0.05). During PEG-catalase effects on FE Na were similar to those observed for Tempol: no change in CON but a marked increase in CKD (P<0.001), resulting in significant interaction (P<0.01). FE K was not affected by either Tempol or PEG-catalase, and neither FE Na nor FE K were affected by vehicle infusion.

### Comparison of changes induced by Tempol, PEG-catalase and vehicle

**Figure 7:**
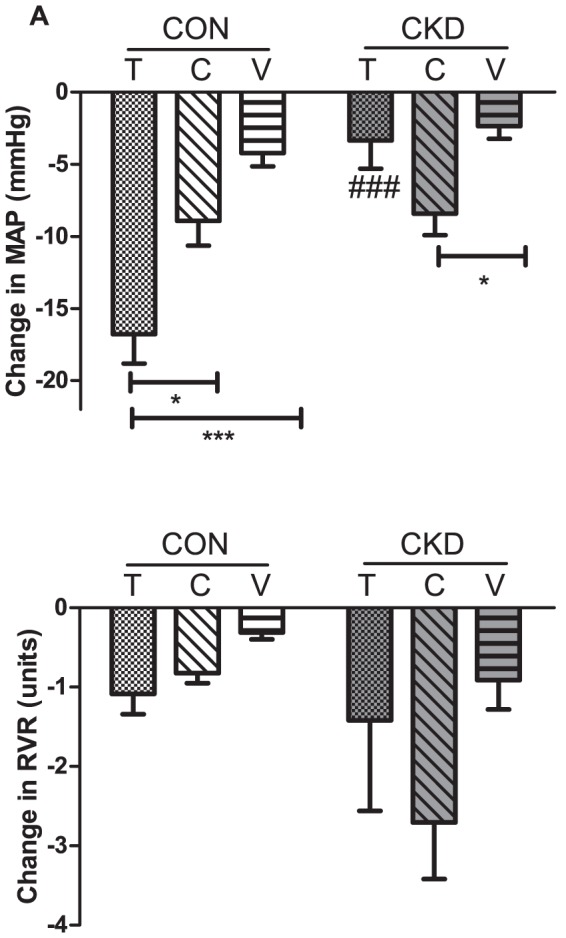
Changes in MAP (panel A) and RVR (panel B) in CON (white bars) and CKD rats (grey bars) during infusion of Tempol (T, bars with squares), PEG-catalase (C, bars with stripes) and vehicle (V, bars with horizontal lines). Mean ± SEM. P CKD vs. CON = 0.0011, P Interventions = 0.0028, P Interaction = 0.0014, for panel A; P CKD vs. CON  =  NS, P Interventions  =  NS, P Interaction  =  NS for panel B). Tukey post-hoc test for comparison between groups: ### P<0.01 vs. CON. Between groups: *P<0.05. **P<0.01 ***P<0.001.

Figure 7 depicts the changes in MAP and RVR after acute administration of Tempol, PEG-catalase or vehicle in CON and CKD rats. For change in MAP an overall effect of all three interventions was observed (P<0.001) as well as interaction (P<0.01, Fig. 7A). Tempol infusion decreased MAP by nearly 15 mmHg in CON rats (P<0.001) but MAP remained unchanged in CKD rats when compared with the change caused by vehicle infusion in the same condition. However, when comparing change in MAP caused by PEG-catalase administration, the opposite was observed: MAP decreased slightly in CON rats but was significantly lower in CKD rats (P<0.05) when compared to change caused by vehicle infusion in the same condition. Changes in RVR were not significantly different (Fig. 7B). One-hour acute infusion of Tempol or PEG-catalase in terminal setting did not cause any changes in the renal expression of RAS and VEGF-A genes or in tyrosine hydroxylase staining in comparison to vehicle infusion in both CON and CKD (Supplemental Table S1 and Supplemental Figure S1).

### Comparison of TBARS excretion induced by Tempol, PEG-catalase or vehicle

**Figure 8:**
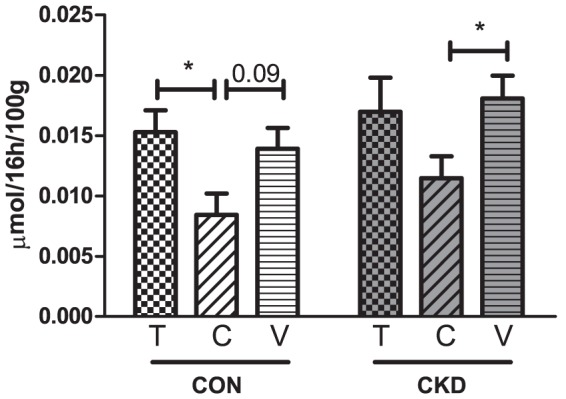
16h TBARS excretion in CON rats (white bars) and CKD rats (grey bars) after intravenous administration of Tempol (T, bars with squares), PEG-catalase (C, bars with stripes) or vehicle (V, bars with horizontal lines). Mean ± SEM. P CON vs. CKD  =  NS; P Interventions = 0.003; P Interaction  =  NS. Tukey post hoc test for comparison between groups: *P<0.05.

Intravenous administration of Tempol did not affect excretion of TBARS in CON and CKD groups compared to vehicle, whereas PEG-catalase decreased TBARS excretion in CKD group (P<0.05) and showed a trend to decrease in CON group compared to vehicle (P = 0.09) (Fig. 8).

## Discussion

The main novel finding of this study is that in established CKD, MAP and RVR do not depend on ROS. This was demonstrated by the failure to alter MAP in CKD rats by acute scavenging of superoxide with Tempol. Reducing H_2_O_2_ with PEG-catalase did not normalize MAP in CKD rats. Furthermore, in CKD rats, Tempol had no effect on TBARS excretion while PEG-catalase reduced it.

Parameters of oxidative stress are increased and antioxidant enzyme activities are decreased in patients with various degrees of CKD [38]–[40]. Important endogenous antioxidant enzymes are SOD(s) that convert superoxide to H_2_O_2_, which is in turn disposed of by two other enzymes, catalase and glutathione peroxidase. In experimental CKD a marked down-regulation of hepatic and renal cytoplasmic and mitochondrial SOD was found as well as down-regulation of renal catalase and glutathione peroxidase protein abundance and catalase activity [41], [42].

### Effect of Tempol and PEG-catalase on MAP

In CKD rat models chronic Tempol administration only ameliorated hypertension for 10–14 days after nephrectomy [20], [21]. Our data suggests that in long-term experimental CKD, once hypertension is established, other mechanisms contribute to its maintenance. Because Tempol caused a marked decrease in MAP in CON but not in CKD rats, maintenance of hypertension in our model of CKD appears not to depend on superoxide. Although Tempol infusion reduces superoxide levels, it results in accumulation of H_2_O_2_ that might serve as an important hypertensive factor [7] and has been reported to induce renal vasoconstriction [43], [44]. The lack of antihypertensive effects of Tempol might be explained by the need of a fully functional system of other (non-SOD) antioxidant enzymes to drive the H_2_O_2_ generated from superoxide dismutation to CO_2_ and H_2_O. In contrast to Tempol, we found that acute administration of PEG-catalase did decrease MAP in CKD. However, MAP was not normalized to control levels in response to PEG-catalase, suggesting that H_2_O_2_ is not solely responsible for hypertension in established CKD. Increased production of ROS can reduce the availability of vasodilators such as nitric oxide (NO), which can lead to functional NO deficiency and thus contribute to maintenance of hypertension. Indeed, CKD rats in the present study showed a tendency to lower levels of urinary NOx excretion vs. CON rats. However, VEGF-A gene expression and endothelial cell staining, although both clearly reduced in CKD rats, were not affected acutely by Tempol and PEG-catalase. Other factors than oxidative stress that can affect the blood pressure are RAS and the sympathetic nervous system. We found no changes in either gene expression of AT1, ACE1 or renin (Supplemental Table S1) or in detection of sympathetic nerves between treatment groups (Supplemental Figure S1). Thus, at least these levels of expression, these known regulators of blood pressure and renal perfusion were not acutely affected by Tempol and PEG-catalase.

### Effect of Tempol and PEG-catalase on RVR

Tempol and PEG-catalase had limited effects on RVR in CKD suggesting that renal resistance vessels are not sensitive to renal vasoconstrictor effects of ROS in this model. We found no other reports on renal hemodynamics during acute treatment with either Tempol or PEG-catalase in rats with established CKD. Because we chose for a systemic intravenous rather than renal intra-arterial administration of Tempol and PEG-catalase we cannot evaluate their direct effects on the kidney. One might hypothesize that ROS-mediated vasoconstriction in the extrarenal circulation contributes to hypertension in established, long-term CKD. Although increased myogenic tone preceded structural vascular changes and hypertension in rats with CKD induced by renal mass reduction [45], ultimately, loss of myogenic response of the mesenteric arteries was observed [46]. Moreover, segments of the mesenteric arteries from CKD rats incubated with Tempol and PEG-catalase showed a significant increase rather than decrease in myogenic constriction suggesting that superoxide and H_2_O_2_ may be involved in pathological loss of the myogenic response [47].

### Effect of Tempol and PEG-catalase on TBARS excretion

Tempol showed no effect on urinary TBARS excretion in neither CON nor CKD rats suggesting that it failed to reduce oxidative stress in both groups. Similar to the effect on MAP in the acute experiment, PEG-catalase reduced TBARS excretion in both CON and CKD. This once again suggests that oxidative stress is not the main force driving maintenance of hypertension in this established model of CKD.

### Effect of Tempol and PEG-catalase on FE Na

A striking finding in this study is that FE Na in CKD rats was increased by both Tempol and PEG-catalase in comparison to CON rats suggesting that excessive ROS modulate natriuresis. In agreement with our observation, it has been demonstrated that ROS decreases sodium excretion [48]. It has been shown that ROS have multiple anti-natriuretic tubular actions [49]. Our data suggests, as indicated by the increase of FE Na, that Tempol and PEG-catalase decreased tubular reabsorption. The observation that both Tempol and PEG-catalase had no effects on MAP and RBF suggests that, in this model of CKD, they acted mainly via tubular mechanisms and thus can only affect BP indirectly and hence slowly. We observed a time-dependent reduction of GFR in all groups. However, relative to baseline, the reduction in the vehicle control group was smaller than the one observed in the Tempol and PEG-catalase control groups. Moreover, no significant difference was observed between the baseline and vehicle measurements in the CKD groups.

In conclusion, in the current study we show that in established CKD MAP and RVR did not depend more on ROS than in CON. Our findings suggest that antioxidant therapy in experimental CKD, although it can prevent the increase in BP in early stages, might not be effective in reducing BP once CKD is established.

## Supporting Information

Figure S1 Immunohistochemical labeling of renal tissue for tyrosine hydroxylase (TH) in CON rats (first row) and CKD rats (second row) to detect sympathetic nerves (green, white arrows).
(DOCX)

Table S1 Gene expression of renin, AT1, ACE1 and VEGF-A in CON and CKD rats (first cohort), after intravenous infusion of with Tempol, PEG-catalase or vehicle in terminal setting. Data are presented as log fold change relative to the calibrator (vehicle treated animals in CON and CKD groups). Means ± SEM.
(DOCX)
