# Correction: Maintenance of Hypertensive Hemodynamics Does Not Depend on ROS in Established Experimental Chronic Kidney Disease

**DOI:** 10.1371/journal.pone.0099476

**Published:** 2014-06-04

**Authors:** The PLOS ONE Staff

An error occurred while the manuscript was being prepared for publication. Figures 2 and 3 do not appear in color in the PDF version of the paper, making the images difficult to read.

Please see the corrected Figure 2 here.

**Figure 2:**
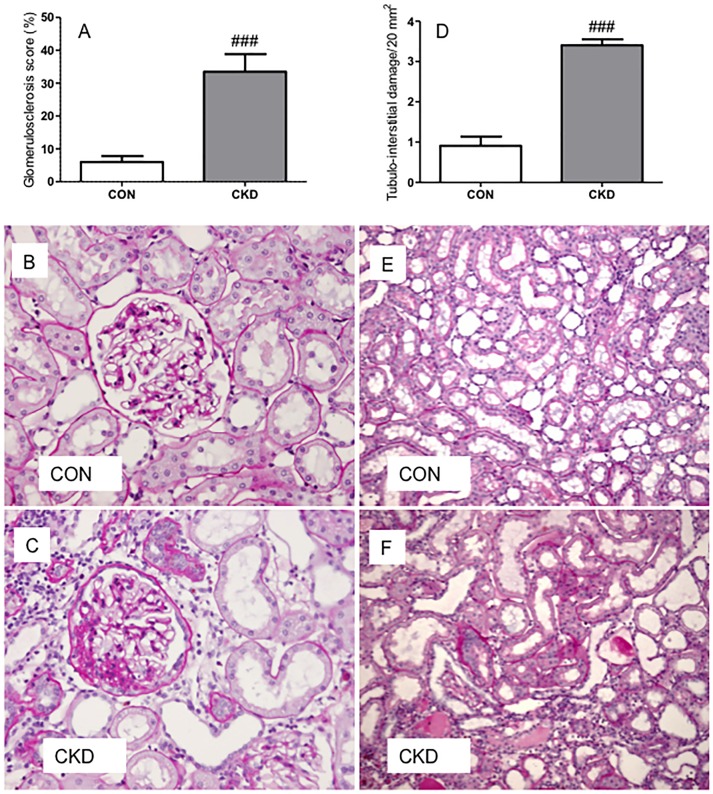
Bilateral ablation (C and F) induced more glomerulosclerosis (panel A) and tubulo-interstitial damage (panel D) in CKD rats compared to controls (B and E) on PAS-stained sections. Means ± SEM. Unpaired t-test: ###P<0.001 vs. CON.

Please see the corrected Figure 3 here.

**Figure 3:**
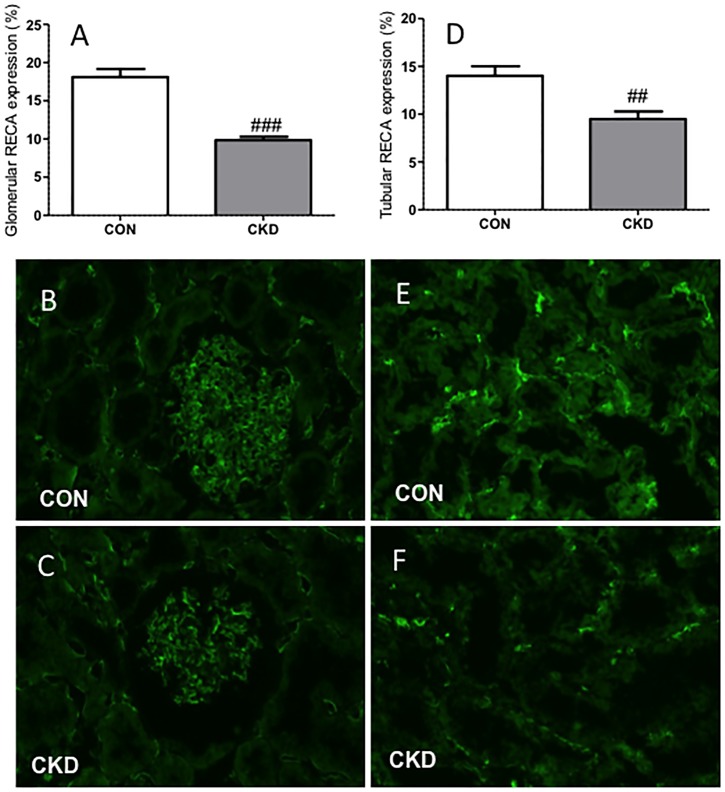
Less (RECA)+ pixels (green) were found in CKD rats compared to CON rats in both glomeruli (panel A) and tubular fields (panel D). Immunohistochemical labeling is shown in CON rats (panels B and E) and in CKD rats (panels C and F). Means ± SEM. Unpaired t-test: ###P<0.001; ##P<0.01 vs. CON.
