# Supplementary material for: Maintenance of Hypertensive Hemodynamics Does Not Depend on ROS in Established Experimental Chronic Kidney Disease
Source: PLoS One. 2014 Feb 12;9(2):e88596. doi: 10.1371/journal.pone.0088596 (PMC3922946; doi:10.1371/journal.pone.0088596)
Supplement: Figure S1 — Immunohistochemical labeling of renal tissue for tyrosine hydroxylase (TH) in CON rats (first row) and CKD rats (second row) to detect sympathetic nerves (green, white arrows). (DOCX) [file pone.0088596.s001.docx]

**Supplemental Figure 1.** Immunohistochemical labeling of renal tissue for tyrosine hydroxylase (TH) in CON rats (first row) and CKD rats (second row) to detect sympathetic nerves (green, white arrows).


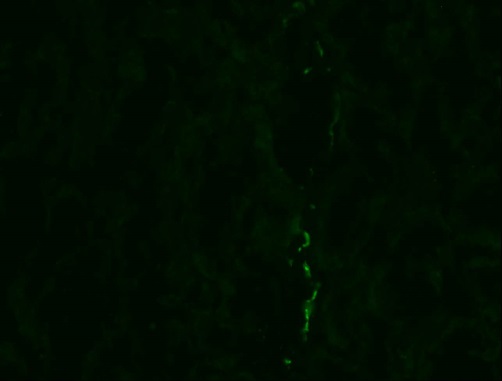

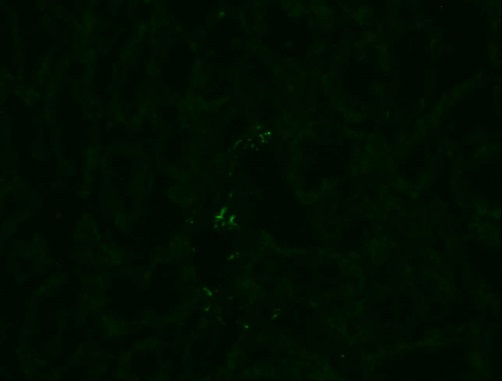

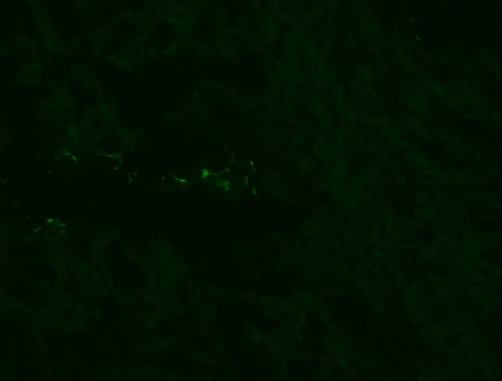


CON, Tempol


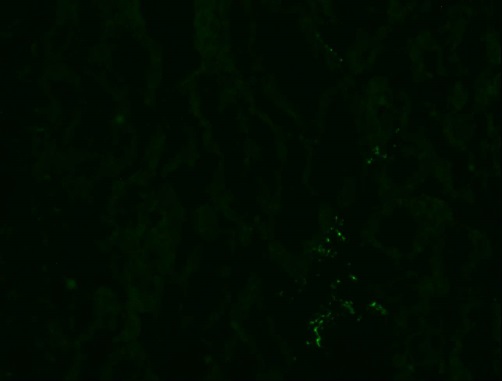


CON, PEG-catalase

CON, vehicle


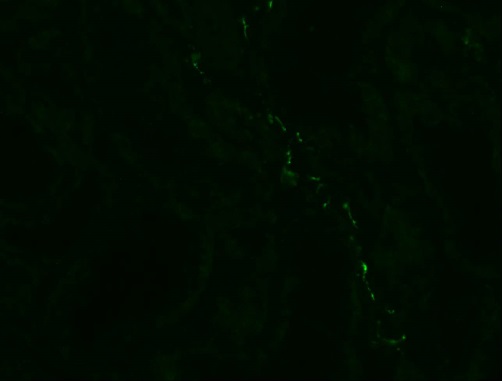


CKD, Tempol


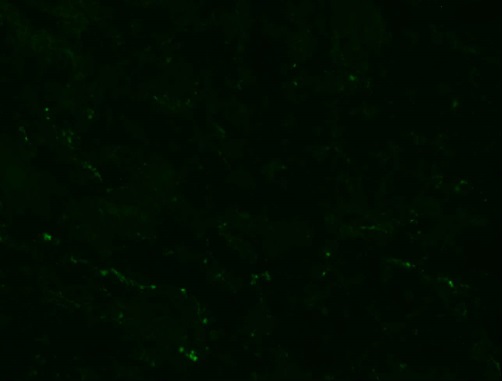


CKD, PEG-catalase

CKD, vehicle
