# Supplementary material for: Maintenance of Hypertensive Hemodynamics Does Not Depend on ROS in Established Experimental Chronic Kidney Disease
Source: PLoS One. 2014 Feb 12;9(2):e88596. doi: 10.1371/journal.pone.0088596 (PMC3922946; doi:10.1371/journal.pone.0088596)
Supplement: Table S1 — Gene expression of renin, AT1, ACE1 and VEGF-A in CON and CKD rats (first cohort), after intravenous infusion of with Tempol, PEG-catalase or vehicle in terminal setting. Data are presented as log fold change relative to the calibrator (vehicle treated animals in CON and CKD groups). Means ± SEM. (DOCX) [file pone.0088596.s002.docx]

**Supplemental table 1.** Gene expression of renin, AT1, ACE1 and VEGF-A in CON and CKD rats (first cohort), after intravenous infusion of with Tempol, PEG-catalase or vehicle in terminal setting. Data are presented as log fold change relative to the calibrator (vehicle treated animals in CON and CKD groups).Means ± SEM.

|  | **CON** | | | **CKD** | | |
| --- | --- | --- | --- | --- | --- | --- |
|  | **Tempol** | **PEG-catalase** | **Vehicle** | **Tempol** | **PEG-catalase** | **Vehicle** |
| renin | 0.36± 0.47 | -0.04 ±0.32 | 0.0 ± 0.37 | 0.08 ± 0.38 | 0.88 ± 0.31 | 0.0 ± 0.35 |
| AT1 | -0.27± 0.14 | 0.0 ±0.26 | 0.0 ± 0.15 | 0.19 ± 0.24 | 0.47 ± 0.16 | 0.0 ± 0.24 |
| ACE1 | 0.02 ±0.29 | 0.12± 0.24 | 0.0 ± 0.17 | -0.47 ± 0.38 | -0.29 ± 0.30 | 0.0 ± 0.59 |
| VEGF-A | -0.43± 0.14 | -0.05 ± 0.25 | 0.0 ± 0.10 | 0.62 ± 0.15 | 0.92 ± 0.28 | 0.0 ± 0.46 |
